# Deepening into the suitability of using pre-trained models of ImageNet against a lightweight convolutional neural network in medical imaging: an experimental study

**DOI:** 10.7717/peerj-cs.715

**Published:** 2021-09-28

**Authors:** Laith Alzubaidi, Ye Duan, Ayad Al-Dujaili, Ibraheem Kasim Ibraheem, Ahmed H. Alkenani, Jose Santamaría, Mohammed A. Fadhel, Omran Al-Shamma, Jinglan Zhang

**Affiliations:** 1 School of Computer Science, Queensland University of Technology, Brisbane, Queensland, Australia; 2 AlNidhal Campus, University of Information Technology & Communications, Baghdad, Baghdad, Iraq; 3 Faculty of Electrical Engineering & Computer Science, University of Missouri - Columbia, Columbia, Missouri, United States; 4 Electrical Engineering Technical College, Middle Technical University, Baghdad, Baghdad, Iraq; 5 Department of Electrical Engineering, College of Engineering, University of Baghdad, Baghdad, Baghdad, Iraq; 6 The Australian E-Health Research Centre, CSIRO, Brisbane, Queensland, Australia; 7 Department of Computer Science, University of Jaén, Jaén, Jaén, Spain; 8 College of Computer Science and Information Technology, University of Sumer, Rafia, Thi Qar, Iraq

**Keywords:** Transfer learning, Deep learning, ImageNet, Convolutional neural network, Medical imaging

## Abstract

Transfer learning (TL) has been widely utilized to address the lack of training data for deep learning models. Specifically, one of the most popular uses of TL has been for the pre-trained models of the ImageNet dataset. Nevertheless, although these pre-trained models have shown an effective performance in several domains of application, those models may not offer significant benefits in all instances when dealing with medical imaging scenarios. Such models were designed to classify a thousand classes of natural images. There are fundamental differences between these models and those dealing with medical imaging tasks regarding learned features. Most medical imaging applications range from two to ten different classes, where we suspect that it would not be necessary to employ deeper learning models. This paper investigates such a hypothesis and develops an experimental study to examine the corresponding conclusions about this issue. The lightweight convolutional neural network (CNN) model and the pre-trained models have been evaluated using three different medical imaging datasets. We have trained the lightweight CNN model and the pre-trained models with two scenarios which are with a small number of images once and a large number of images once again. Surprisingly, it has been found that the lightweight model trained from scratch achieved a more competitive performance when compared to the pre-trained model. More importantly, the lightweight CNN model can be successfully trained and tested using basic computational tools and provide high-quality results, specifically when using medical imaging datasets.

## Introduction

Recently, transfer learning (TL) (Pan & Yang, 2009; Zhuang et al., 2020) has revealed itself as an essential tool (in association with growth in the field of deep learning) in dealing with multiple challenging classification tasks. Nevertheless, the current trend in medical imaging is to employ pre-trained models, such as Xception (Chollet, 2017) and ResNet (He et al., 2016), by using natural image datasets like ImageNet (Krizhevsky, Sutskever & Hinton, 2012). However, it is well known that the weights of these models need to be accurately fine-tuned to improve performance, specifically when facing complex scenarios within medical imaging.

The previous approach has been widely adopted in the majority of medical specialties within medical imaging, such as ophthalmology (ResNet and Inception-V3 models trained on retinal fundus images (Abràmoff et al., 2016; De Fauw et al., 2018; Gulshan et al., 2016) and radiology (DenseNet and ResNet models trained using chest X-rays; Rajpurkar et al., 2017; Wang et al., 2017). Ophthalmology research has resulted in broad clinical deployment (Van Der Heijden et al., 2018) and finally achieved the approval of the FDA (Topol, 2019). More applications involve obtaining quality human embryos for IVF practices (Khosravi et al., 2018), recognizing dermatology levels from skin cancer images (Thomas et al., 2021), and achieving early detection of Alzheimer’s disease (Chitradevi & Prabha, 2020).

As stated, there is an increasing interest in using TL in the field of medical imaging (Morid, Borjali & Del Fiol, 2020; Shinde et al., 2021; Karimi, Warfield & Gholipour, 2021). However, its pros and cons have not been adequately addressed in state-of-the-art systems (SoTA). More specifically, in the natural image setting (He, Girshick & Dollár, 2019; Kornblith, Shlens & Le, 2019; Geirhos et al., 2018; Wen et al., 2021; Alzubaidi et al., 2020b, 2021a), the current work-based TL has faced several common dilemmas. For instance, He, Girshick & Dollár (2019) showed that TL did not perform well in all the addressed instances and did not provide an apparent reason for such results. On the contrary, Kornblith, Shlens & Le (2019) concluded that the previous approach was more general than considering the pre-trained features. Furthermore, some ambiguity is still apparent when examining medical imaging scenarios using TL.

As previously explained, TL is usually applied using a convolutional neural network (CNN) model from ImageNet and its pre-trained weights. The model is then fine-tuned to tackle the target task efficiently. Medical image diagnosis and ImageNet vary significantly in several aspects. The tasks in medical imaging begin with capturing images of a wide section of the region of interest. Next, pathology identification is achieved by recognizing the differences in local textures within images. For example, diabetic retinopathy and micro-aneurysms are usually identified by small red dots in retinal fundus images (Shankar et al., 2020). Additionally, indications of pneumonia and consolidation are identified by local white opaque patches in chest X-rays. ImageNet can often identify trivial prominent objects present in the image, a clear example of its applicability to natural image datasets. However, it is unclear whether the level of ImageNet’s feature re-utilization conveys a significant improvement of the identification process in medical imaging scenarios. It was recently proven that the performance of the pre-trained models declined when they were employed for images such as chest X-rays and brain MRI, as these models have not been trained on gray-scale images (Cherti & Jitsev, 2021).

At their inception, pre-trained ImageNet models were designed to classify one thousand natural images, thus making it necessary to adjust a significant number of parameters accurately. Today, datasets used in medical imaging use fewer images. ImageNet datasets can range from hundreds to thousands and even a hundred thousand in rare cases. When tackling medical imaging scenarios, such as diabetic foot ulcers, skin cancer, and sickle cell anemia, there are between two and ten (Alzubaidi et al., 2020c; Pacheco & Krohling, 2021; Alzubaidi et al., 2020a). Therefore, the experiential hypothesis concludes that it is unnecessary to use highly trained models for medical imaging tasks.

This paper analyzes the benefits of using the TL of pre-trained ImageNet models for medical imaging tasks. For comparison, a hybrid lightweight CNN model was designed that combines recent advancements in the field, including parallel convolutional layers, residual connection, and global average pooling. In the experimental study, two pre-trained ImageNet models (ResNet50 and Xception) and the lightweight model were used to examine three different datasets: diabetic foot ulcers, sickle cell anemia, and skin cancer. Finally, the study compared this domain TL in medical tasks, to the TL of the pre-trained models. Based on the above-mentioned information, we compared the pretrained models and the proposed model as listed in Table 1.

**Table 1:** Comparison of the pre-trained models to the lightweight CNN model.

Feature	The pre-trained models	The Lightweight CNN model
Depth	Very deep	Shallow and lightweight
Input size	Fix input size (such as: 224 × 224)	Flexible (such as: 512 × 512)
Computational tools	Required high computational tools	Required low computational tools
Number of classes	Designed for 1,000 classes	Designed for 2–10 classes
Type of image	Natural images, colour images	Colorful, gray, large size images
Details of image	General object	Details important
Training time	long	short
Transfer learning	Natural images as a source	Same-domain TL can be used

The structure of the paper is as follows. “Brief Overview” introduces a summary of the application to be investigated and its datasets. The pre-trained ImageNet models and TL are introduced in “The Pre-trained ImageNet Models”. “The Proposed Model” details the proposed lightweight CNN model, and “Experimental Results” presents the experimental study carried out in this work. Finally, “Conclusions” ends with some conclusions and future research.

## Brief overview

This section presents a brief introduction to the practical applications addressed and the specific datasets used for each application tackled in this paper. This section does not intend to provide a broad review of the SoTA of deep learning and medical imaging. However, the reader can investigate this topic more in-depth by reading the author’s previous paper (Alzubaidi et al., 2021c).

### Diabetic foot ulcer

One of the complications of diabetes is diabetic foot ulcers (DFU) that can result in the removal of a foot or limb (Alzubaidi et al., 2020c). Commonly, the DFU is situated on the heel, and symptoms often include dry cracks, leg pain, foot swelling, skin temperature differences, and skin color changes. The cost of DFU treatment is usually high, and mortality rates increase if the condition is not treated at an early stage. The critical parameters for predicting the DFU’s amputation risk or healing potential are detecting and diagnosing the infection or areas of ischemia (Tulloch, Zamani & Akrami, 2020). Chronic diabetes in the human body causes poor blood circulation that in turn causes ischemia (Everett & Mathioudakis, 2018). Therefore, palpating the pulses of blood flow in the foot can help discover ischemia (Amin et al., 2020), and poor foot reperfusion, which could develop into a DFU infection, shows the ischemia visually (Peter-Riesch, 2016). From the researchers’ point of view, detecting the DFU is the objective, as (a) the appearance of DFU can change in terms of location, shape, and size, (b) resemblances between intra- and inter-class variations, and (c) lighting conditions. It should be noted that there are comprehensive, in-depth medical studies relating to leg blood vessels, blood tests, bacteriological studies, and physical examinations. However, these tests and resources are often inaccessible worldwide.

This paper used a combination of two DFU datasets: (Goyal et al., 2018; Alzubaidi et al., 2020c). The first one (Goyal et al., 2018) contained 1,610 patches taken from foot images. These patches were divided into two classes: normal, with 641 patches, and abnormal, with 969 patches. The second dataset (Alzubaidi et al., 2020c) contained 1,477 patches taken from foot images, with 742 classified as normal and 735 abnormal, to provide a total of 3,087 patches.

### Sickle cell anemia

Red blood cells (RBC) play a vital role in the gassy exchange between living tissue and the external environment. The protein in the RBC that carries oxygen throughout the body is known as hemoglobin (Deshpande, Gite & Aluvalu, 2021; Fadhel, Humaidi & Oleiwi, 2017; Rakshit & Bhowmik, 2013), and directs all life after the age of 6 weeks. Hemoglobin consists of two beta and two alpha chains (Fadhel, Humaidi & Oleiwi, 2017). If each parent donates an abnormal copy of the hemoglobin gene, the offspring will contract sickle cell anemia. More specifically, sickle hemoglobin (HbS) will replace healthy hemoglobin (HbA) (Rakshit & Bhowmik, 2013). If HbS replace half the HbA, the individual will possess a single abnormal gene or sickle cell traits (Rakshit & Bhowmik, 2013). The life span of a sickle cell is 10–20 days, while the life span of a healthy RBC is 120 days (Rakshit & Bhowmik, 2013).

The process of combining hemoglobin S with a deoxygenated molecule is called hemoglobin polymerization and causes the RBC to look sickle-shaped (Das et al., 2019). The cell morphology plays a significant role in classifying the patient’s clinical state (Das et al., 2019). In the field of biomedicine, cell segmentation and accurate counting become challenging tasks due to the complicated nature of the cells (Das et al., 2019). Overlapping and touching cells have a crucial impact on automated detection, and accurate categorization, as clear separation between cells is required (Das et al., 2019; Bushra & Shobana, 2021). The existence of varying intensity, noise, and the differentiated signal strength of lesion cells, further complicates medical image classification and segmentation (Bushra & Shobana, 2021). Ellipticity, cell texture, circularity, elongation, form factor, area, size, shape, and location are all characteristics that assist in efficient classification and segmentation (Bushra & Shobana, 2021; Petrović et al., 2020).

This paper used two datasets, namely dataset1 and dataset3 (Gonzalez-Hidalgo et al., 2014; Alzubaidi et al., 2020a). Dataset1 (Gonzalez-Hidalgo et al., 2014) was the erythrocytesIDB dataset, comprising 626 images of individual cells labeled as circular (202), elongated (211), or other (213), all sized at 80 × 80 pixels. Dataset3 (Alzubaidi et al., 2020a) contained 726 individual patches, again sized at 80 × 80 pixels. The patches were categorized into three classes: circular cells or normal erythrocytes (292), elongated cells (sickle cell anemia) (201), and other cells (233). The total dataset was made up of 1,352 patches.

### Skin cancer

Skin cancers, malignant melanoma, and non-melanoma incidence rates have recently increased globally (Leiter, Keim & Garbe, 2020; Schadendorf et al., 2018). It is the most common malignancy in the white population, and among skin cancer types, malignant melanoma (MM) is the deadliest worldwide. It should be noted that death and incidence rates vary widely between countries. Annually, 0.7% of all cancer deaths are caused by MM, representing around 55,000 people (Schadendorf et al., 2018). Therefore, early detection is a significant factor in improving the survival rate and successful treatment for melanoma patients (Mahbod et al., 2020; Alzubaidi et al., 2021a).

Due to MM’s morphological patterns, specialist clinical inspection correctly diagnoses melanoma in 65–80% of cases, (Whiteman, Green & Olsen, 2016; Schadendorf et al., 2018). The four parameters of asymmetry, border, color, and diameter (known as the ABCD rule) are frequently used to assess a lesion (Chatterjee et al., 2021). For clinically atypical presentations, illumination and magnification are usually used alongside dermoscopy as supportive imaging techniques to improve the visibility of sub-surface structures, leading to a reduction in screening errors and improving melanoma detection (Tromme et al., 2012). The accuracy of melanoma diagnosis can be improved up to 50% with dermoscopy compared with a purely visual inspection, depending on the dermatologist’s experience (Kelati et al., 2017). In individuals with atypical clinical presentations, diagnostic accuracy is significantly changed (Phillips et al., 2020). Therefore, the idea of developing fully or semi-automated computer-aided diagnostic systems (CAD) (Brinker et al., 2018; Okuboyejo & Olugbara, 2018) for screening programs or use as an independent second opinion has gained significant interest. The CAD systems can be based on advanced machine learning concepts or classical image processing (Oliveira et al., 2018). Currently, skin lesions are usually classified in four stages: pre-processing, segmentation, feature extraction, and training (to decide) (Oliveira et al., 2018). Several factors have significant impact on the performance of these methods. For instance, artifacts like bubbles and skin hairs, or lesions with fuzzy borders, complicate the segmentation task. Other complicating factors include varying light conditions and hand-crafted features (Oliveira et al., 2018).

This paper adopted the SIIM-ISIC 2020 dataset (available at: https://www.kaggle.com/c/siim-isic-melanoma-classification), comprising 33,000 skin lesion samples categorized as benign or malignant. The dataset had only 584 images in the malignant class, and 10,000 of the remaining benign images were used. In addressing this data imbalance, images of malignant skin cancers were collected from previous datasets, and additionally, image augmentation techniques were performed, including cropping, rotation to different angles, and brightening. The total number of images of both classes was 20,040. The images were divided into 80% for training and 20% for testing.

## The pre-trained imagenet models

In medical image analysis and machine vision, the most commonly used type of network is a CNN (Al-Timemy et al., 2021). This network includes several layers: input (one layer), convolution (one or more layers), pooling (many layers), and one fully connected layer (Alzubaidi et al., 2021c). Several CNN architectures have been proposed in the last 10 years (Alzubaidi et al., 2021c, Morid, Borjali & Del Fiol, 2020). This section describes the ImageNet, the CNN architectures, and TL used in this paper.

### ImageNet

Since 2010, one of the famous annual competitions in the field of large-scale object recognition has been the ImageNet Large-Scale Visual Recognition Competition (ILSVRC) (Krizhevsky, Sutskever & Hinton, 2012). The ImageNet dataset, comprising more than 15 million labeled images, is one of several datasets involved in the competition (Krizhevsky, Sutskever & Hinton, 2012). Several CNN models, some described briefly in the following sections, employ the ImageNet dataset to classify images into their matching classes very successfully. More details about these models can be found in Reference (Alzubaidi et al., 2021c). ImageNet is the most widely used dataset for medical image analysis-based TL, as Cheplygina, de Bruijne & Pluim (2019) reported in a recent review. The ResNet and Xception models have proved to be most accurate on ImageNet (Khan et al., 2020; Alzubaidi et al., 2021c). Thus, we have chosen to use them in this paper.

### ResNet

Vanishing gradients and accuracy saturation are two issues that arise from inserting additional layers into CNN models. The backbone of the ResNet model that targets solving these issues is known as residual learning (He et al., 2016). Before ResNet, the CNN model learned features after passing over the convolutional layer with different abstraction levels. Instead of feature-learning, ResNet learns residuals. For each convolutional layer, the residual results from subtracting the learned features from the input, using an identity shortcut connections idea (*i.e*., the input layer is connected to several layers after that). Different versions of ResNet are available based on the number of layers, such as ResNet101, ResNet50, and ResNet34.

### Xception

Xception is an improved version of Inception-V3 (Chollet, 2017). It stands for extreme inception since, in the training process, it employs depth-wise separable convolution to include image channel dimension and image spatial dimension individually. Compared to Inception-V3, Xception has a little improved performance on ImageNet and often has a similar number of parameters.

### Transfer learning

**Figure 1:**
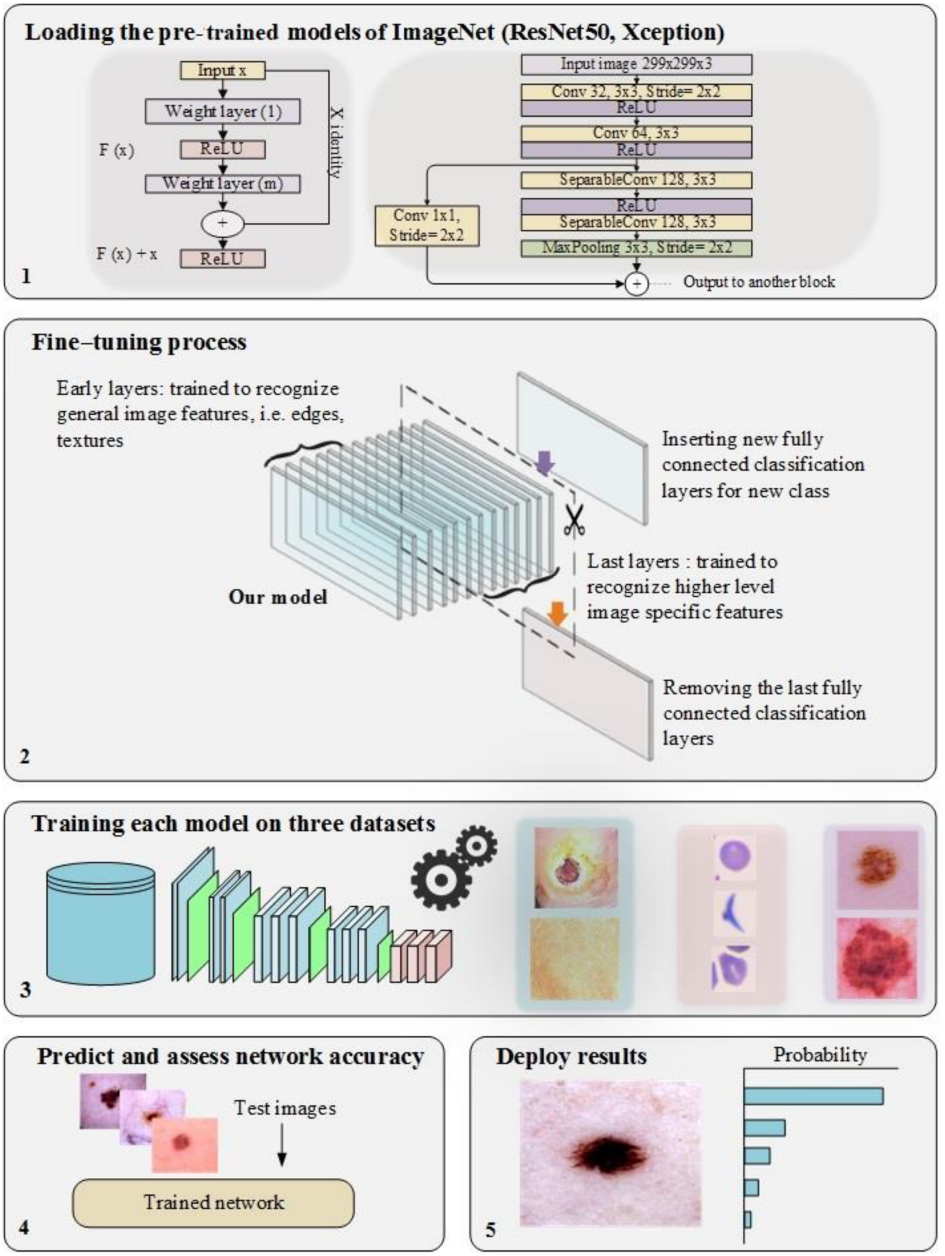
The workflow of the pre-trained models, including the fine-tuning process.

In training CNN models, the most common issue is the shortage of a significant number of labeled datasets (Zhou, Zhang & Zhang, 2019; Zhuang et al., 2020; Alzubaidi et al., 2020a). Medical image analysis problems can be solved using TL, such as transferring the weights (learned parameters) of a model (well-trained CNN) trained on a sizable dataset like ImageNet. Typically, a well-trained CNN model is used to achieve TL by either freezing or fine-tuning the convolution layers and training the fully connected layer from scratch (using the medical dataset of the issue under consideration) (Alzubaidi et al., 2021b). As previously mentioned, the learned features of ImageNet’s pre-trained models are entirely different from medical imaging features, and therefore, the benefit of TL from these models can be limited. Two of the most popular pre-trained models (ResNet50 and Xception) were fine-tuned, as shown in Fig. 1, to validate this hypothesis.

## The proposed model

### The proposed model’s architecture

**Figure 2:**
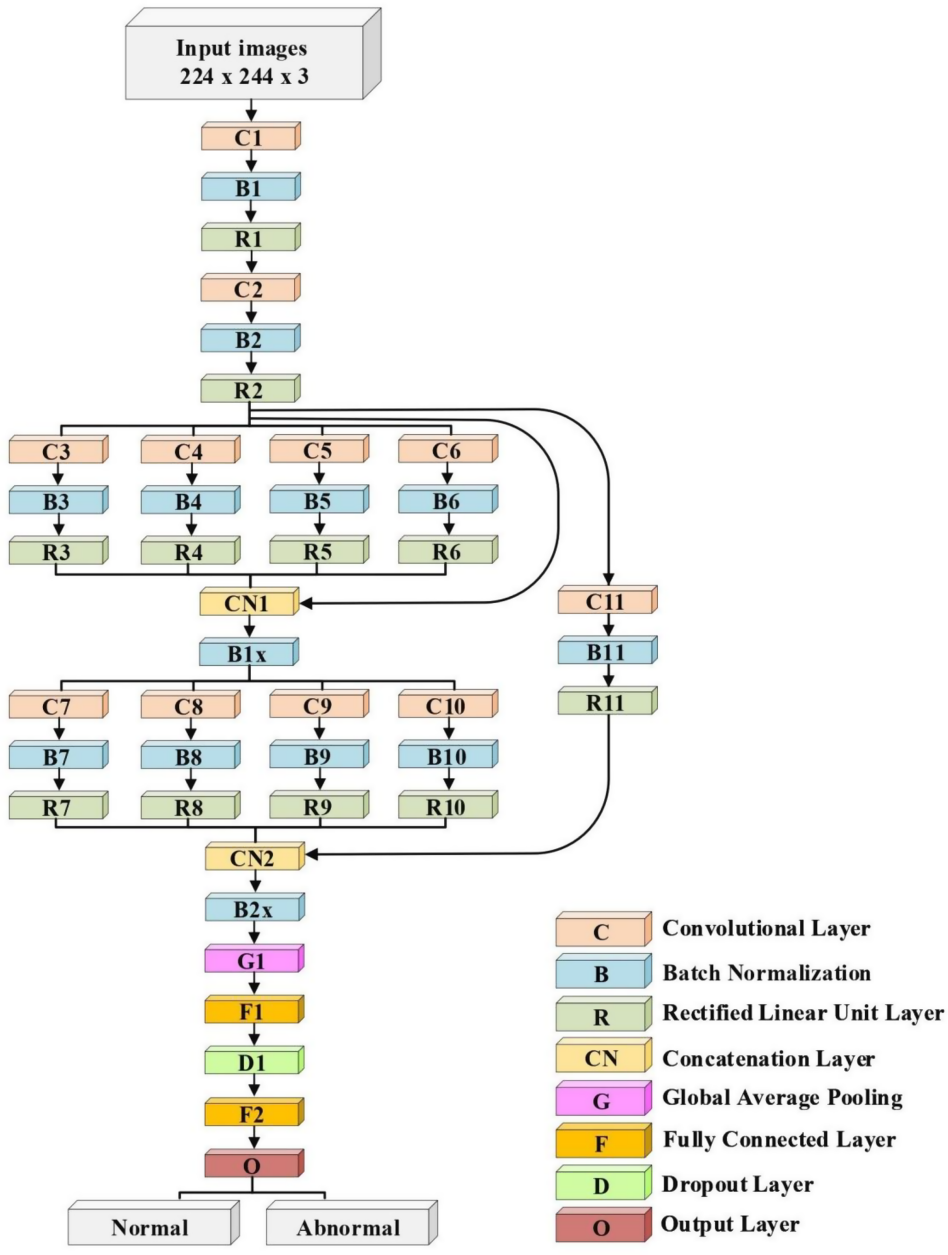
The architecture of the proposed model.

A lightweight model that can be compared with the performance of the pre-trained models ResNet50 and Xception has been designed. The model consists of several different components. The first is that the model starts with two convolutional layers sequenced to reduce the size of the input image. The first layer has a filter size of 3 × 3, and the second one has a filter size of 5 × 5. Every convolutional layer in the model is followed by a batch normalization layer and a rectified linear unit (ReLU). The output of these two layers is pushed to two blocks of parallel convolutional layers, with each block consisting of four convolutional layers working in parallel. The four convolutional layers have different filter sizes: 1 × 1, 3 × 3, 5 × 5, and 7 × 7. The variance in filter size helps to capture small and large features. The output of the four layers concatenates in the concatenation layer. Residual connections have been utilized in the two blocks to have better feature representation.The output of the two blocks is dimensionally reduced by the global average pooling layer, followed by two fully connected layers with a dropout layer between them. The model architecture ends with a softmax function for classification. Table 2 and Fig. 2 illustrate the architecture of the proposed model. Due to the employment of a ReLU, which does not squeeze the input, and the shallow architecture of our model, the gradient vanishing issue cannot happen. The employment of dropout, batch normalization, and global average pooling help the model to avoid overfitting issues. The model dodges from employing the max or average pooling layers after each convolutional layer to prevent feature loss and keeps only global average pooling at the end. This layer takes the average of the whole input and produces one vector value.

**Table 2:** The model's architecture. C = Convolutional layer, B = Batch normalization layer, R = Rectified linear unit layer, CN = Concatenation layer, G = Global average pooling layer, D = Dropoutlayer, and F = Fully connected layer.

Layer Number	Filter Size (FS) and Stride (S)	Activations
Input layer	–	224 × 224 × 3
C1, B1, R1	FS = 3 × 3, S = 1	224 × 224 × 32
C2, B2, R2	FS = 5 × 5, S = 2	112 × 112 × 32
C3, B3, R3	FS = 1 × 1, S = 1	112 × 112 × 32
C4, B4, R4	FS = 3 × 3, S = 1	112 × 112 × 32
C5, B5, R5	FS = 5 × 5, S = 1	112 × 112 × 32
C6, B6, R6	FS = 7 × 7, S = 1	112 × 112 × 32
CN1	Five inputs	112 × 112 × 160
B1x	Batch Normalization Layer	112 × 112 × 160
C7, B7, R7	FS = 1 × 1, S = 2	56 × 56 × 64
C8, B8, R8	FS = 3 × 3, S = 2	56 × 56 × 64
C9, B9, R9	FS = 5 × 5, S = 2	56 × 56 × 64
C10, B10, R10	FS = 7 × 7, S = 2	56 × 56 × 64
C11, B11, R11	FS = 3 × 3, S = 2	56 × 56 × 32
CN2	Five inputs	56 × 56 × 228
B2x	Batch Normalization Layer	56 × 56 × 228
G1	–	1 × 1 × 228
F1	200 FC	1 × 1 × 200
D1	Dropout layer with learning rate:0.5	1 × 1 × 200
F2	2 FC	1 × 1 × 2
O (Softmax function)	Normal, Abnormal	1 × 1 × 2

### Training the proposed model

The parameters adopted in training were stochastic gradient descent with momentum set to 0.9. The mini-batch size was 32, and MaxEpochs was 100, with a learning rate initially set to 0.001. The experiments were performed using Matlab 2020 as software, on a processor with Intel (R) Core TM i7-5829K CPU, 3.30 GHz, 32 GB RAM, and 16 GB GPU.

The proposed model was trained with different datasets.

**Figure 3:**
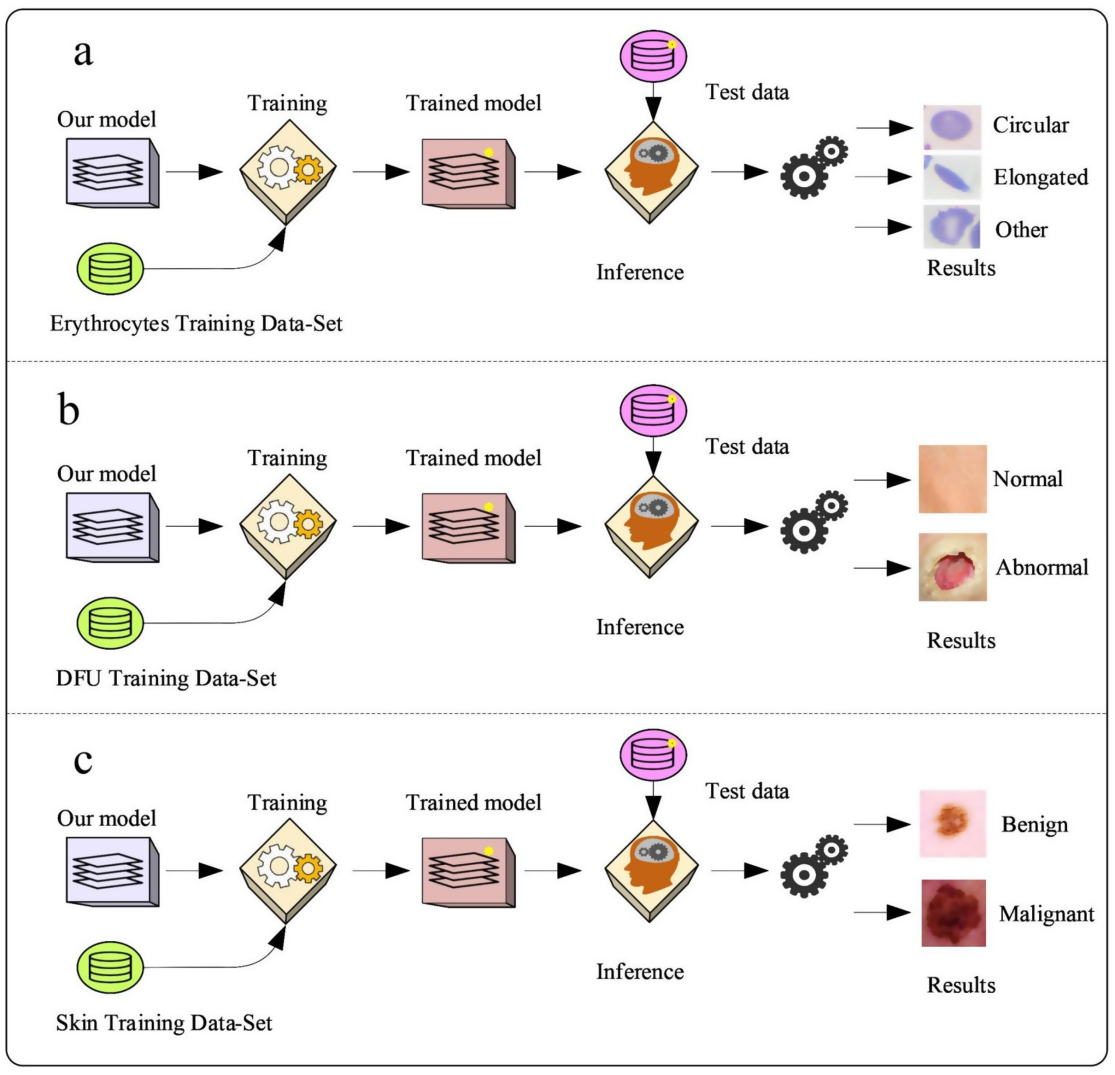
The workflow of the proposed model with three datasets.

**Figure 4:**
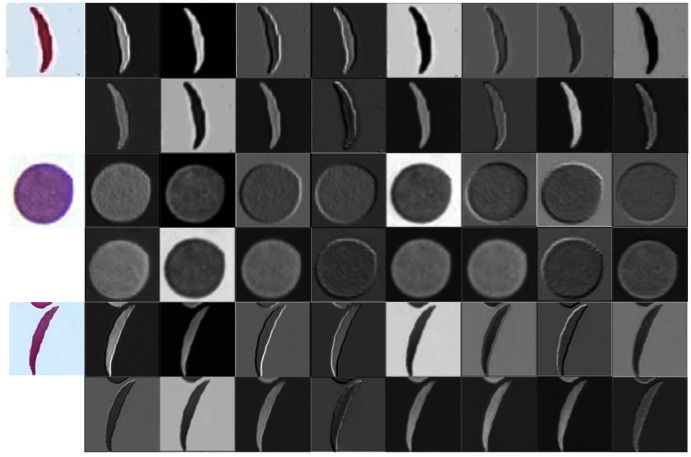
Learned filters from the first convolution layer of the model trained on erythrocyte images.

**Figure 5:**
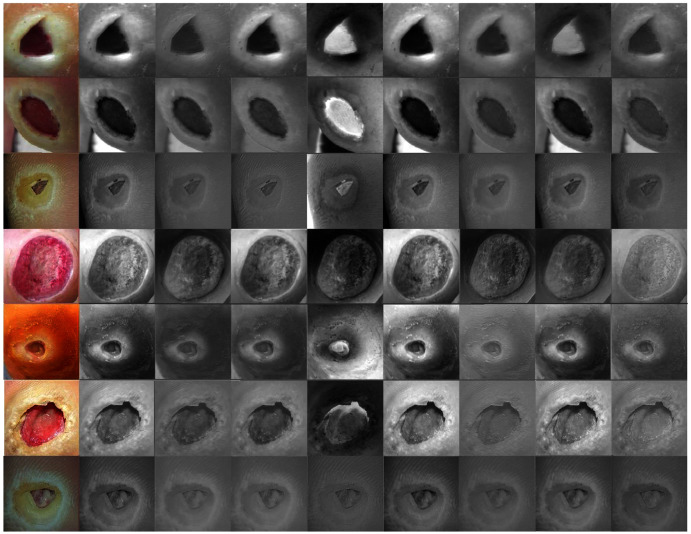
Learned filters from the first convolution layer of the model trained on foot images.

**Figure 6:**
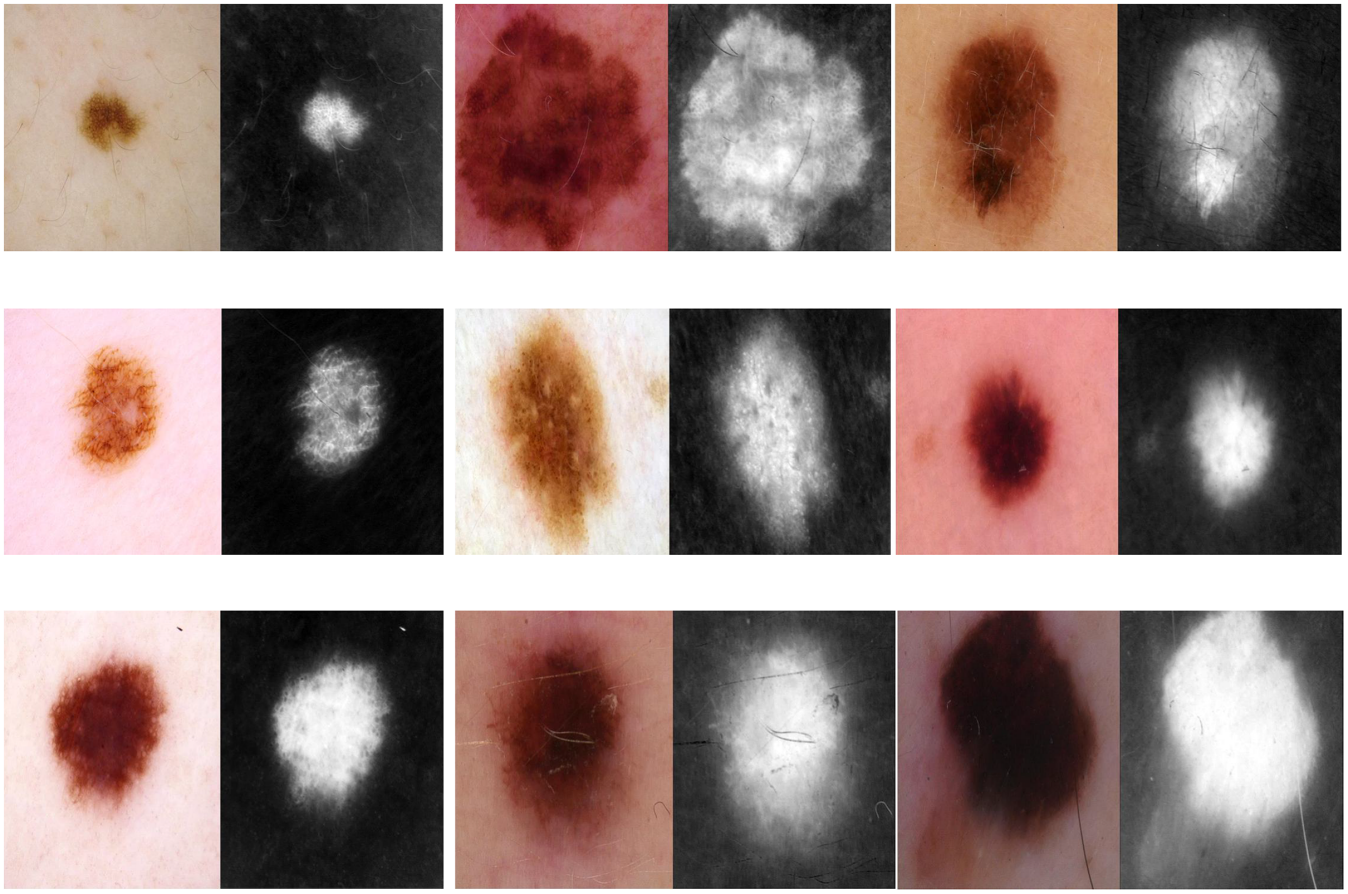
Learned filters from the first convolution layer of the model trained on skin images.

1. Erythrocyte images were classified into three grades, circular, elongated, or other, as shown in Fig. 3A. The input size of the proposed model was set to 80 × 80 to match the input images. We trained the proposed model with the original dataset, in addition to augmented images, using several data augmentation techniques, including rotation on different angles, flipping, zooming, cropping, brightness, and contrast. Figure 4 shows the learned filter of the first convolutional layer.
2. Images of foot patches were classified into two grades, normal or abnormal, as shown in Fig. 3B. The input size of the proposed model was set to 224 × 224 to match the input images. The proposed model was trained with two scenarios of DUF task. First, the model was trained with 1,000 of the original dataset patches, then with 10,000, plus augmented images using several data augmentation techniques including rotation on different angles, flipping, zooming, cropping, brightness, and contrast.
  Figure 5 shows the learned filter of the first convolutional layer trained with the second scenario.
3. Skin image patches were classified into two grades, benign and malignant, as shown in Fig. 3C. The input size of the proposed model was set to 500 ×375 to match the input images. The proposed model was trained with two scenarios of skin cancer. First, the model was trained with 2,000 patches from the original dataset, then with 20.000. Augmented images were only used for one class, as mentioned above, to resolve imbalanced data. Figure 6 shows the learned filter of the first convolutional layer, trained with the second scenario.

The same training parameters, datasets, and training scenarios were used to train the pre-trained ResNet50 and Xception models.

### Feature extraction in the proposed model

**Figure 7:**
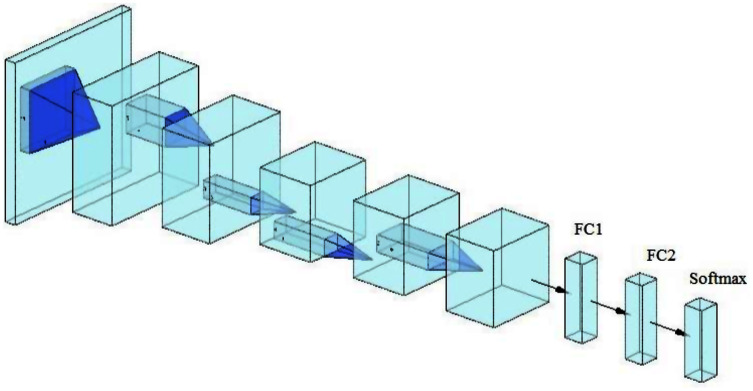
Convolutional neuronal network (CNN).

Two automatic steps of CNNs are feature extraction and classification (Tabares-Soto et al., 2021, Al-yousif et al., 2021). One of the main factors of performing an excellent classification is the extracted features. Therefore, to demonstrate the model’s effectiveness in feature extraction, the extracted features were utilized for training the support vector machine (SVM) classifier and testing it with the same test set. The SVM classifier often shows the best results in many tasks, especially in binary classification (Alzubaidi et al., 2020c, Mohammadi et al., 2021; Albashish et al., 2021). Therefore, we employed it in this paper. The last fully connected layer in (FC2, Fig. 7) depicts the final layer before the classifier. This layer had higher-level features, which were used to train the SVM. This experiment was applied to sickle cell anemia and DFU classification tasks. The SVM was used as DFU classification is binary. The sickle cell anemia classification had three classes; therefore, a multiclass model was used to support the vector machines. The Matlab function (fitcecoc) was employed to implement multi-SVM.

### Transfer learning in the proposed model

Recent studies in medical imaging showed that same-domain and in-domain TL obtained comparable or better performance than the pre-trained ImageNet models concerning the reduced number of images used (Alzubaidi et al., 2020b, 2021a). Therefore, a TL experiment was performed on the DFU model, explained as follows:

1. Training the proposed model using numerous images similar to the DFU images. These included 594 images from 15 wound categories (available at: https://github.com/mlaradji/deep-learning-for-wound-care), 2,700 images of various wounds gathered from the internet, and 30,364 images of skin cancer.
2. Fine-tuning the model after it was trained using the images in step 1.
3. Training the pre-trained model from step 2 on the target DFU dataset. For this experiment, more foot-patch images (Alzubaidi et al., 2020b) were added to the previous datasets, giving a total of 4,696 images. The dataset was divided into 55% (adding augmented images) for training, and 45% for testing, as it was beneficial to test the model with a large number of images.
4. Testing the trained model from step 3 with the test set and calculating the evaluation metrics.

## Experimental results

This section is divided as follows: evaluation metrics, results of the DFU, sickle cell anemia, and skin cancer classification tasks, results of the proposed model with SVM, and results of the proposed model with TL.

### Evaluation matrices

The pre-trained models and the proposed model were assessed based on several evaluation metrics, including accuracy, specificity, recall, precision, and F1-score. The evaluation metrics were computed based on the values of TN, TP, FN, and FP. TN and TP were represented as the number of negative and positive instances, respectively, correctly classified. FN and FP were represented as the number of misclassified positive and negative cases, respectively. The equations for each evaluation metric are listed below:

(1) $$Accuracy = \displaystyle{{TP + TN} \over {TP + TN + FP + FN}}$$

(2) $$Specificity = \displaystyle{{TN} \over {FP + TN}}$$

(3) $$Recall = \displaystyle{{TP} \over {TP + FN}}$$

(4) $$Precision = \displaystyle{{TP} \over {TP + FP}}$$

(5) $$F{1_{score}} = 2 \times \displaystyle{{Precision \times Recall} \over {Precision + Recall}}$$

### Results of the DFU classification task

First, the proposed model and the pre-trained models were evaluated based on training with the first scenario, as reported in Table 3.

**Table 3:** Results of the DUF classification task trained with 1,000 images.

Model	Accuracy (%)	Specificity (%)	Recall (%)	Precision (%)	F1-Score (%)
Proposed model	87.6	86.2	88.8	87.3	88.5
Xception	87.4	86.3	88.4	87.3	87.9
ResNet50	88.6	86.4	90.9	87.4	89.0

The Xception model achieved the lowest values of 87.4%, 86.3%, 88.4%, 87.3%, and 87.9% for accuracy, specificity, recall, precision, and F1-Score, respectively. We strongly believe the low performance of the Xception model was due to very deep architecture that required more training data.

With a small difference from the Xception model, the proposed model finished in second place. It achieved an accuracy of 87.6%, specificity 86.2%, recall 88.8%, precision 87.3%, and F1-Score 88.5%.

The ResNet50 obtained the highest results by achieving 88.6%, 86.4%, 90.9%, 87.4%, and 89.0% for accuracy, specificity, recall, precision, and F1-Score, respectively. All models roughly achieved close results.

In the second training scenario, the training data increased to ten times that of the first training scenario. To examine the difference, we evaluated the proposed model and the pre-trained models on a testing set.

**Figure 8:**
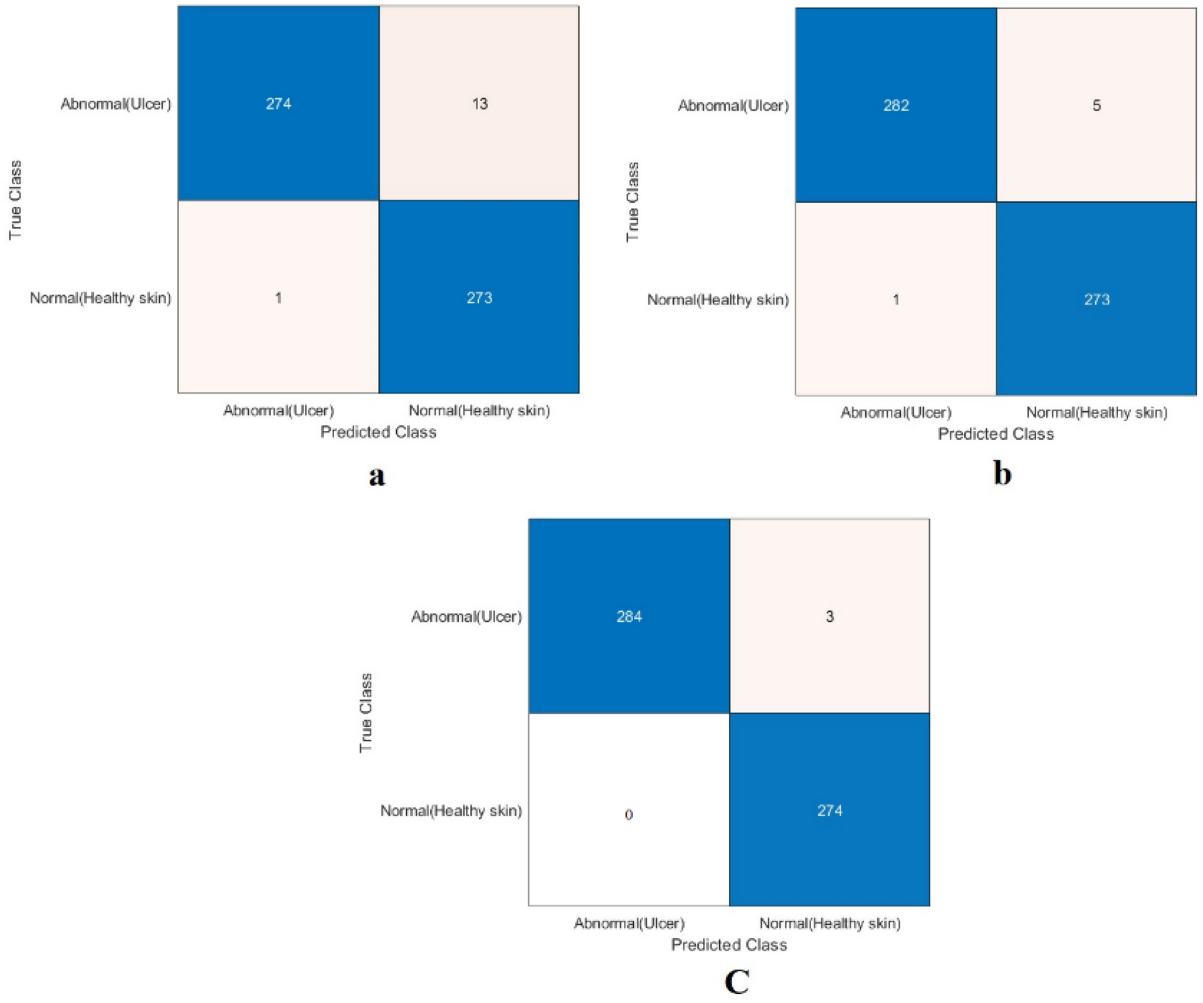
Confusion metrics of the DFU task for (A) the proposed model, (B) Xception, and (C) ResNet50.

The value of TN, TP, FN, and FP were calculated first, as shown in Fig. 8. The proposed model and the pre-trained models were evaluated based on these values, as reported in Table 4. The ResNet50 model achieved the highest values by obtaining 99.4%, 98.9%, 100%, 98.9%, and 99.4% for accuracy, specificity, recall, precision, and F1-Score, respectively. With a small difference from the ResNet50 model, the Xception model came in second place. It achieved an accuracy of 98.9%, specificity 98.2%, recall 99.6%, precision 98.2%, and F1-Score 98.9%. Although the proposed model achieved the lowest results, they were still competitive with the ResNet50 and Xception models. The proposed model obtained 97.5%, 95.5%, 99.6%, 95.4%, and 97.5% for accuracy, specificity, recall, precision, and F1-Score, respectively.

**Table 4:** Results of the DUF classification task trained with 10,000 images.

Model	Accuracy (%)	Specificity (%)	Recall (%)	Precision (%)	F1-Score (%)
Proposed model	97.5	95.5	99.6	95.4	97.5
Xception	98.9	98.2	99.6	98.2	98.9
ResNet50	99.4	98.9	100	98.9	99.4

It is worth mentioning that both the ResNet50 and Xception models were trained by transferring ImageNet’s learned features, while the proposed model was trained from scratch in both training scenarios. The results obtained showed that TL did not offer significant benefits. The simple model, trained from scratch, showed results competitive with the deep learning, pre-trained models. The proposed model’s performance improved with the incremental increase of medical imaging task training data, demonstrating that ImageNet’s TL is of no benefit in medical imaging applications.

### Results of the sickle cell anemia classification task

**Figure 9:**
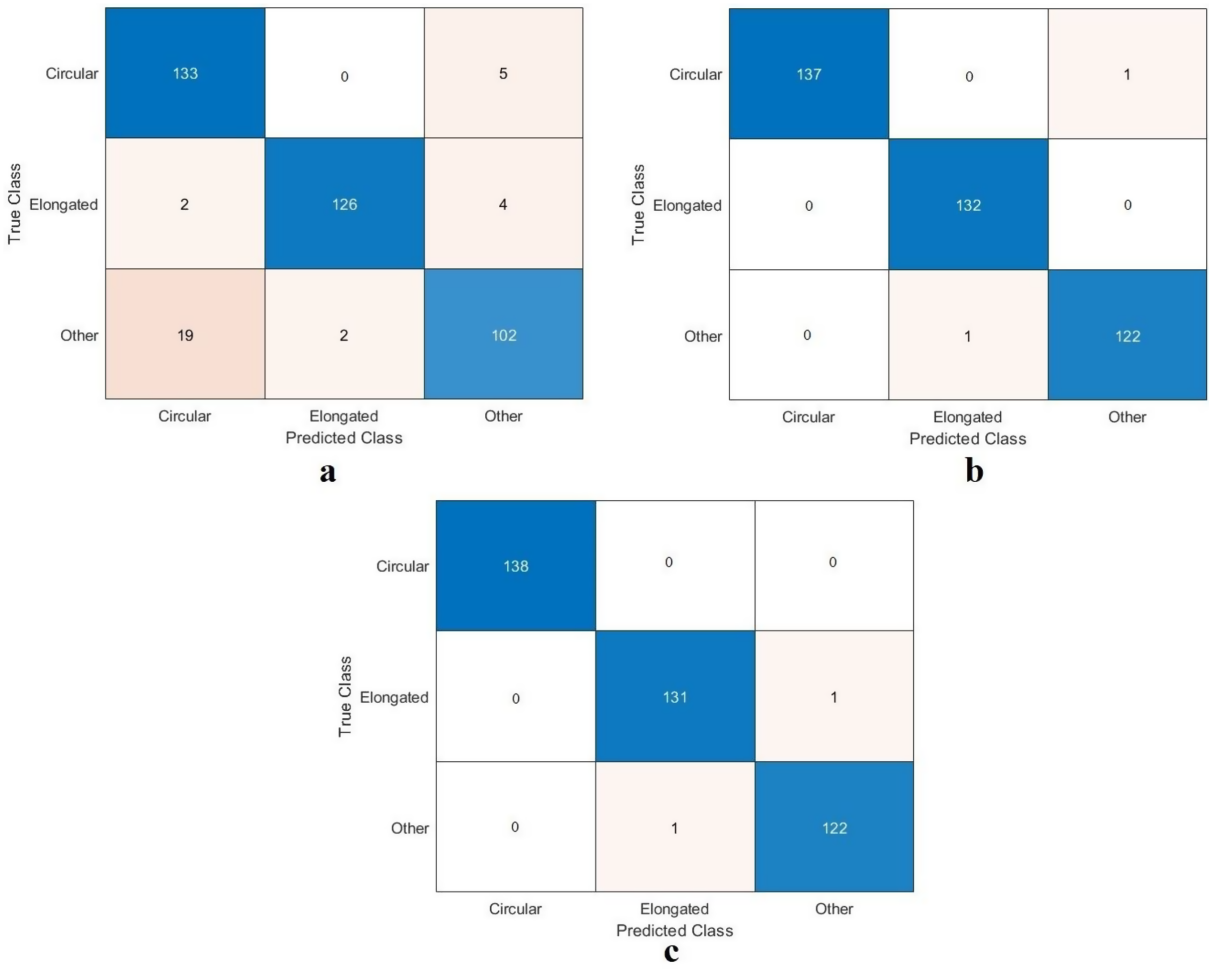
Confusion metrics of the sickle cell anemia task for (A) the proposed model, (B) Xception, and (C) ResNet50.

The values of TN, TP, FN, and FP were calculated, as illustrated in Fig. 9. As with the DFU classification task, the sickle cell anemia task was accomplished with all the models evaluated based on the values, as reported in Table 5. The ResNet50 model again achieved the highest values by obtaining 99.4%, 96.6%, 99.2%, 99.2%, and 99.2% for accuracy, specificity, recall, precision, and F1-Score, respectively. The Xception model accomplished second place, attaining an accuracy of 99.4%, specificity 99.6%, recall 99.1%, precision 99.1%, and an F1-Score of 99.1%. The proposed model obtained 91.8%, 96.6%, 82.9%, 91.8%, and 87.1% for accuracy, specificity, recall, precision, and F1-Score, respectively. Although the proposed model came last, the achieved results were still competitive with the ResNet50 and Xception models. These results were improved using different classifiers as described in Section “Results in the Proposed Model with SVM”, Results of the proposed model with SVM.

**Table 5:** Results of the sickle cell anemia classification task.

Model	Accuracy (%)	Specificity (%)	Recall (%)	Precision (%)	F1-Score (%)
Proposed model	91.8	96.6	82.9	91.8	87.1
Xception	99.4	99.6	99.1	99.1	99.1
ResNet50	99.4	99.6	99.2	99.2	99.2

### Results of the skin cancer classification task

The ResNet50 model achieved the highest values in the previous tasks; therefore, we evaluated the proposed model against the ResNet50 only, as reported in Table 6. Both the proposed and the ResNet50 models trained with scenario one. The proposed model and The ResNet50 had approximately the same results. The proposed model attained values of 87.6%, 86.3%, 88.8%, 87.3%, and 88.0% for accuracy, specificity, recall, precision, and F1-Score, respectively. The ResNet50 achieved accuracy 89.3%, specificity 87.6%, recall 90.9%, precision 88.4%, and F1-Score 89.6%.

**Table 6:** Results of the skin cancer classification task trained with 2,000 images.

Model	Accuracy (%)	Specificity (%)	Recall (%)	Precision (%)	F1-Score (%)
Proposed model	87.6	86.3	88.8	87.3	88.0
ResNet50	89.3	87.6	90.9	88.4	89.6

**Figure 10:**
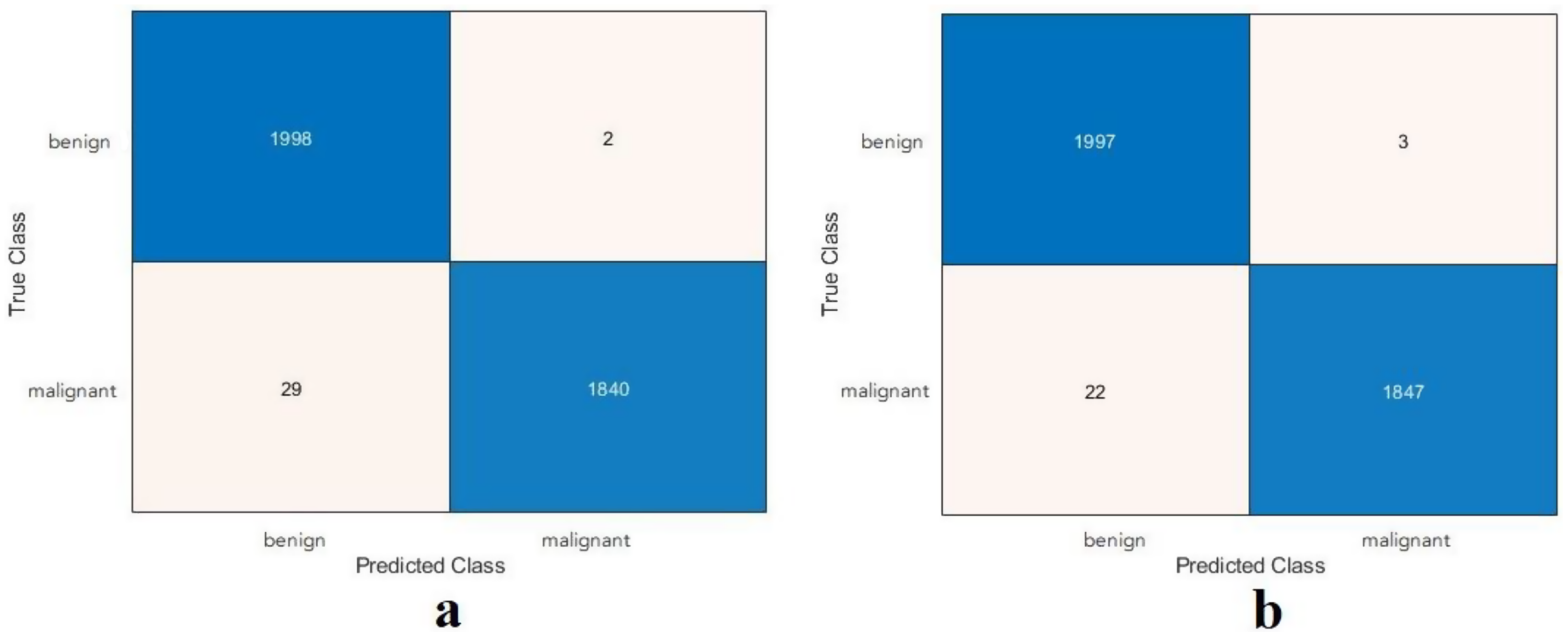
Confusion metrics of the skin cancer task for (A) the proposed model, (B) ResNet50.

Based on the calculated values of TN, TP, FN, and FP, as shown in Fig. 10, the proposed model and the ResNet50 were evaluated based on training with scenario 2. Table 7 reports the results of both models. The proposed model and the ResNet50 had approximately the same results. The proposed model attained values of 99.2%, 98.4%, 99.9%, 98.5%, and 99.2% for accuracy, specificity, recall, precision, and F1-Score, respectively. The ResNet50 model achieved accuracy 99.3%, specificity 99.8%, recall 98.8%, precision 99.8%, and F1-Score 99.3%. Due to the large number of images used in the skin cancer task, the proposed model achieved almost the same results as the ResNet50. Therefore, the TL of ImageNet did not help to enhance the results. The same observation had been drawn from the DFU task and repeated with the skin cancer task, proving that the TL of the ImageNet models did not help.

**Table 7:** Results of the skin cancer classification task trained with 20,000 images.

Model	Accuracy (%)	Specificity (%)	Recall (%)	Precision (%)	F1-Score (%)
Proposed model	99.2	98.4	99.9	98.5	99.2
ResNet50	99.3	99.8	98.8	99.8	99.3

### Results in the proposed model with SVM

**Figure 11:**
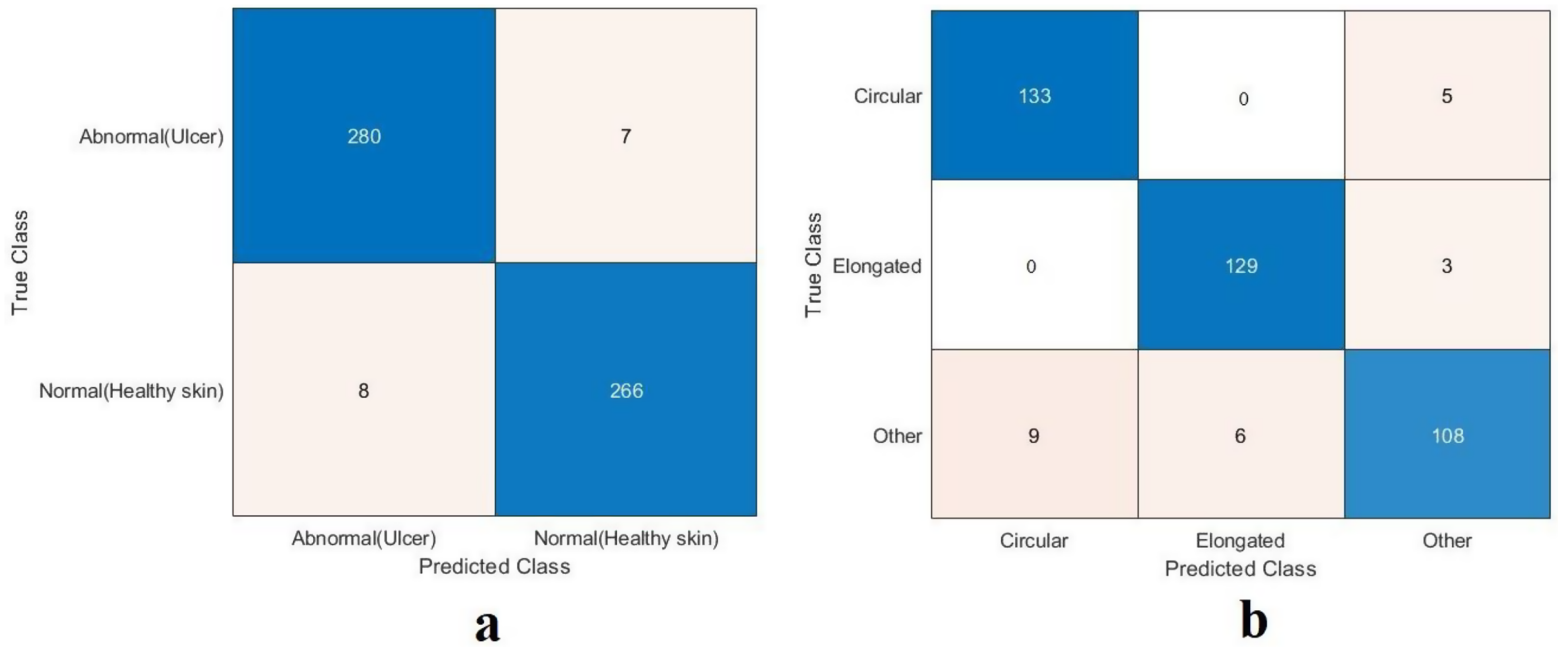
Confusion metrics of the proposed model for (A) DFU and (B) sickle cell anemia.

**Figure 12:**
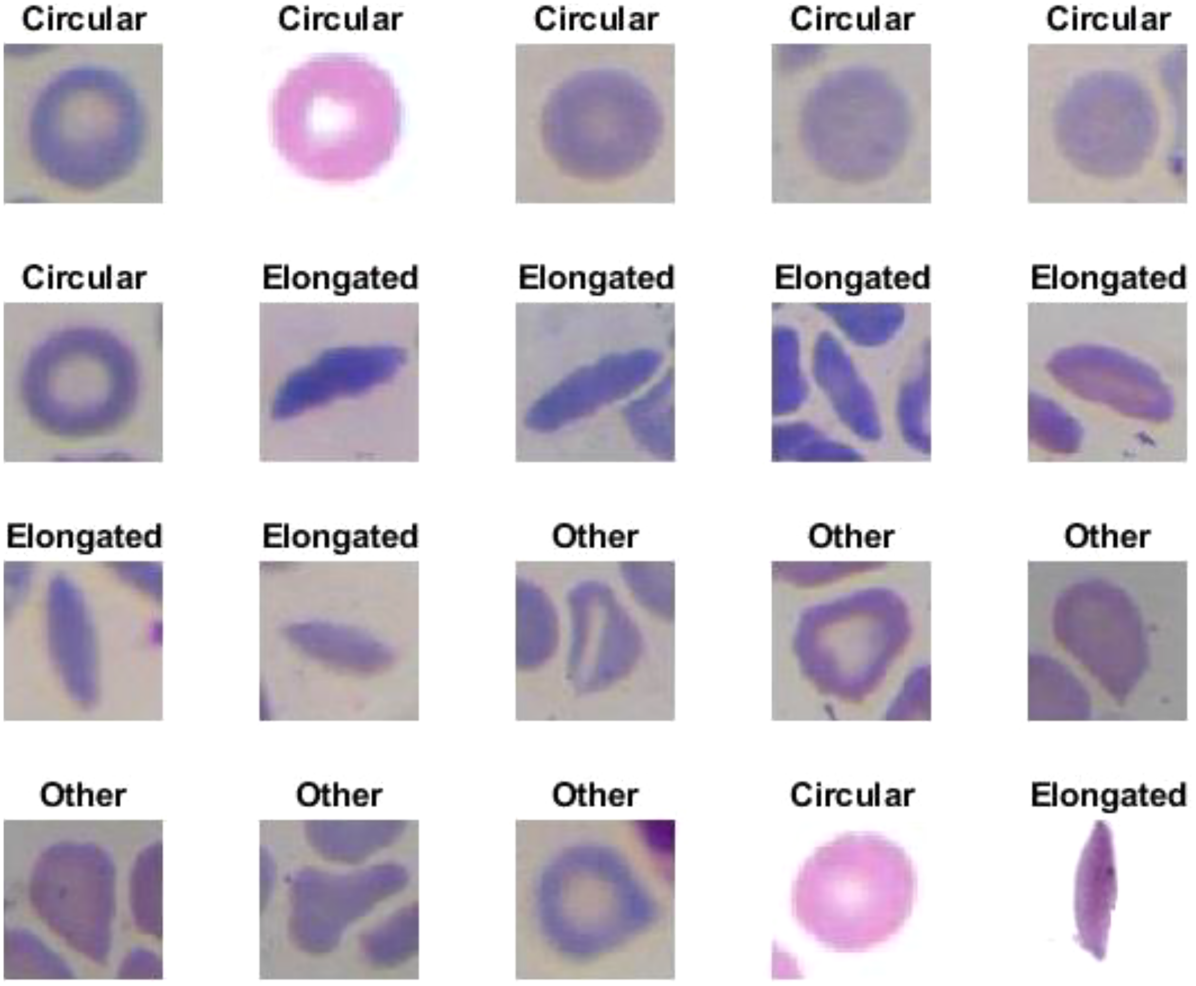
Some of the predictions of erythrocyte samples test set.

The proposed model showed excellent results in feature extraction, proving it to be beneficial, as listed in Table 8. Confusion metrics are shown in Fig. 11. The proposed model results with SVM in the sickle cell anemia task improved on the results shown in Table 3. It achieved 94.2%, 97.1%, 87.8%, 93.1%, and 90.3% for accuracy, specificity, recall, precision, and F1-Score, respectively. However, SVM did not improve the results for the DFU task from those in Table 4. Although similar, these results demonstrated satisfactory extracted features for the proposed model using different classifiers. Some of the predictions of the erythrocyte samples test set are shown in Fig. 12.

**Table 8:** Results of the proposed model with SVM.

Model	Accuracy (%)	Specificity (%)	Recall (%)	Precision (%)	F1-Score (%)
DFU	97.3	97.5	97.1	97.4	97.2
Sickle	94.2	97.1	87.8	93.1	90.3

### Results of the proposed model with transfer learning

**Figure 13:**
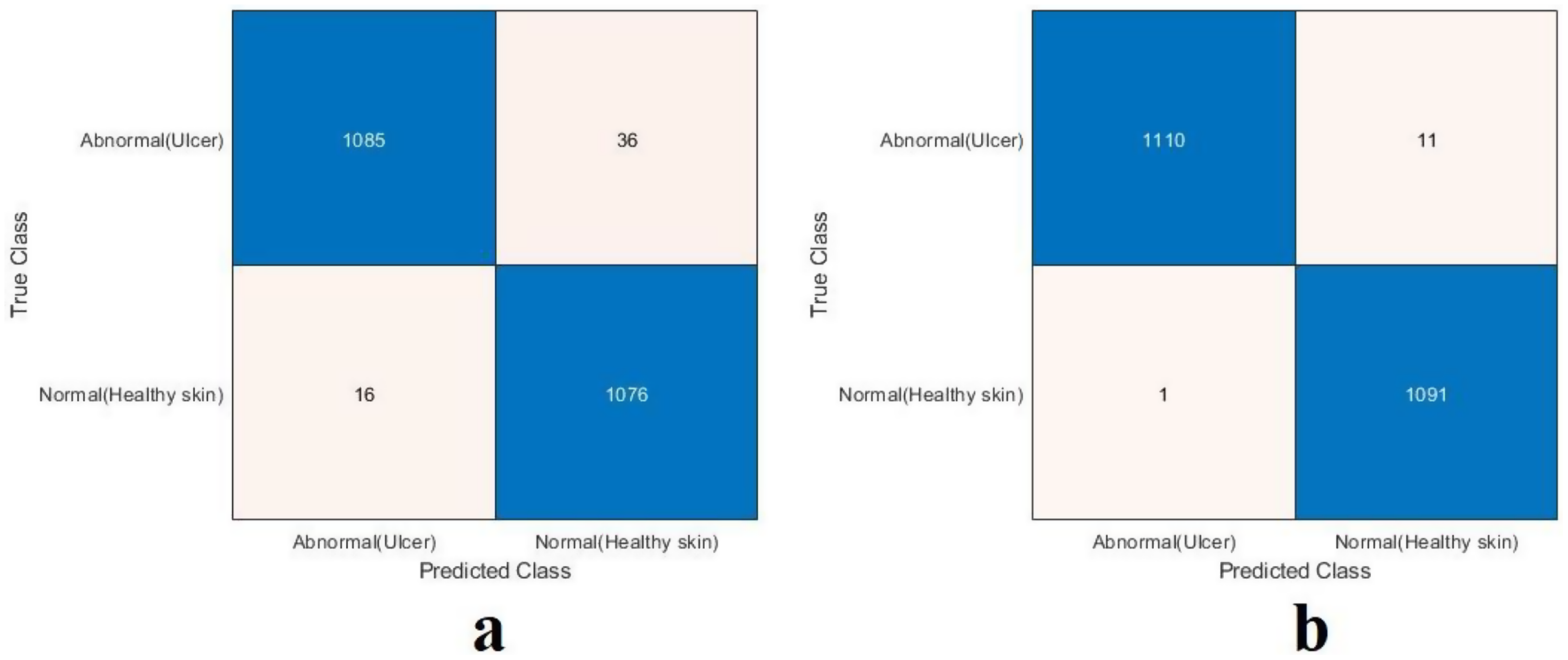
Confusion metrics of the proposed model for (A) training from scratch, (B) training with TL.

**Figure 14:**
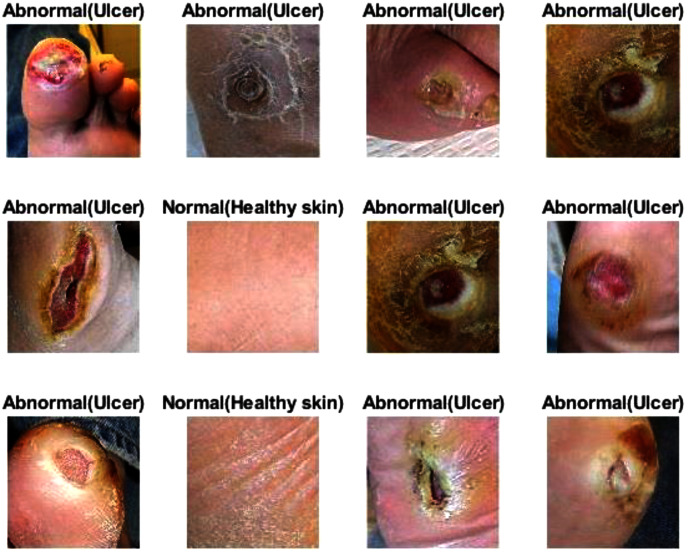
Some of the predictions of the DFU samples test set.

For this section, another DFU dataset was added to the two previous datasets. Figure 13 shows the confusion metrics from before and after TL. Table 9 reports the results of this section. The model was initially evaluated after training from scratch on original images plus augmented images and achieved 97.6%, 96.7%, 98.5%, 96.7%, and 97.6% for accuracy, specificity, recall, precision, and F1-Score, respectively. However, with TL employment, the results improved to an accuracy of 99.5%, specificity 99.1%, recall 99.9%, precision 99.0%, and F1-Score 99.4%. The results achieved with TL are comparative to the ResNet50’s results in Table 4. In an experiment to evaluate the proposed model’s effectiveness in the issue of overfitting, the model was tested on a set of 60 DFU test images provided by the Louisiana State University Health Sciences Center, New Orleans, United States. From the 60 images provided by the Center, 60 patches of DFU were extracted, and 60 patches of normal class were produced from an internet search of foot images. The proposed model tested was the one trained with the TL experiment. It achieved an accuracy of 93.7%, specificity 89.4%, recall 100%, precision 86.6%, and F-score 92.8%. Some of the predictions of the DFU samples test set are shown in Fig. 14. The results proved that our model was effective in robustness against overfitting, as it evaluated unseen images.

**Table 9:** Results of the proposed model with TL.

Model	Accuracy (%)	Specificity (%)	Recall (%)	Precision (%)	F1-Score (%)
From Scratch	97.6	96.7	98.5	96.7	97.6
With TL	99.5	99.1	99.9	99.0	99.4
Unseen Test Set	93.7	89.4	100	86.6	92.8

## Conclusions

This paper has looked in-depth at the benefits of using TL compared with the performance of more general-purpose learning models, such as pre-trained models from ImageNet, specifically when facing challenging medical imaging tasks. In particular, the performance of the proposed lightweight CNN model has been compared with two different pre-trained models: ResNet50 and Xception.

When trained from scratch, the research found that the lightweight model performed nearly as competitively as the pre-trained models from ImageNet. Thus, this research has shown that the benefit of TL from pre-trained ImageNet models is biased to some degree. Additionally, the researchers compared the performance of these models to same-domain TL. It showed that, with a small number of images in the same domain, using the lightweight CNN model could efficiently improve the results compared to an in-depth heavyweight model that considered the transfer of millions of images. Moreover, the research has proved that it is easy to train and test the proposed lightweight CNN model using less demanding computational tools.

Nonetheless, there is still room for improvement, and more research about TL application in medical imaging is needed. Therefore, two different schemes based on TL are currently being developed, which are same-domain and in-domain, to tackle more complex medical imaging tasks. A logical next step for this research is to investigate the benefit of the pre-trained models on ImageNet for segmentation tasks. Moreover, alternative solutions to the TL from ImageNet models for the lack of training data in medical imaging tasks are needed. Therefore, we believe that techniques such as same-domain transfer learning and in-domain transfer learning are worth investigating for medical imaging applications.

## Additional Information and Declarations

### Data Availability

The following information was supplied regarding data availability: The code is available at GitHub: https://github.com/Laith-Alzubaidi/Deepening-into-the-Suitability-of-Using-Pre-trained-Models-of-ImageNet-Against-a-Lightweight-CNN The skin cancer dataset is available at The ISIC 2020 Challenge Dataset: International Skin Imaging Collaboration. SIIM-ISIC 2020 Challenge Dataset. International Skin Imaging Collaboration https://doi.org/10.34970/2020-ds01 (2020). The DFU dataset is available at: http://www2.docm.mmu.ac.uk/STAFF/M.Yap/dataset.php The Sickle Cell dataset is available at the erythrocytesIDB database: http://erythrocytesidb.uib.es/.
